# Methicillin-Resistant Coagulase-Negative *Staphylococci* Carriage is a Protective Factor of Methicillin-Resistant *Staphylococcus Aureus* Nasal Colonization in HIV-Infected Patients: A Cross-Sectional Study

**DOI:** 10.1155/2021/5717413

**Published:** 2021-01-12

**Authors:** Ying Li, Jialing Lin, Linghua Li, Weiping Cai, Jiaping Ye, Suiping He, Wencui Zhang, Ning Liu, Zijun Gong, Xiaohua Ye, Zhenjiang Yao

**Affiliations:** ^1^Department of Epidemiology and Health Statistics, School of Public Health, Guangdong Pharmaceutical University, Guangzhou, China; ^2^School of Population Health, The University of New South Wales, Sydney, Australia; ^3^Department of Infectious Diseases, Guangzhou Eighth People's Hospital, Guangzhou Medical University, Guangzhou, China; ^4^Department of Preventive Health Care, Beihai People's Hospital, Beihai, China

## Abstract

**Background:**

Methicillin-resistant coagulase-negative *Staphylococci* (MRCoNS) is regarded as the repository of mecA gene for methicillin-resistant *Staphylococcus aureus* (MRSA) and may develop methicillin-susceptible *Staphylococcus aureus* (MSSA) to MRSA. Therefore, we aimed to explore whether MRCoNS carriage is a risk factor of MRSA colonization. Phenotypic characteristics were performed to further assess the associations between MRSA and MRCoNS.

**Methods:**

This cross-sectional study was conducted in Guangzhou, China. Participants completed a questionnaire and provided a nasal swab for further analysis. The risk factors of MRSA colonization were analyzed using nonconditional logistic regression models. The phenotypic characteristics between MRSA and MRCoNS were compared by Chi-square test.

**Results:**

Among the 1001 HIV-infected patients, a total of 119 (11.89%) participants were positive for MRSA, and 34.45% (41/119) of all MRSA carriers were positive for MRCoNS. We found MRCoNS carriage was a protective factor of MRSA colonization (adjusted odds ratio = 0.59, 95% confidence interval: 0.38–0.91). A significant difference in the proportions of antibiotic resistance between MRSA and MRCoNS isolates was found except for penicillin, clindamycin, tetracycline, and teicoplanin. The main STs and CC types of MRSA isolates in this population were ST188 (15.1%) and CC59 (17.6%), respectively.

**Conclusions:**

HIV-infected patients remain a highly vulnerable population for MRSA colonization. Though who carried MRCoNS is less likely to have MRSA colonization, similarity of some antibiotic resistance between MRSA and MRCoNS was found in this study. Regular surveillance on the colonization and antibiotic patterns of MRSA and MRCoNS is still necessary.

## 1. Introduction

As a common cause of severe infections, methicillin-resistant *Staphylococcus aureus* (MRSA) is still widespread in both health facilities and the community [1, 2]. Owing to the compromised immune system, HIV-infected patients are more vulnerable to MRSA colonization than other populations [3, 4]. Over the past decade, the prevalence of MRSA isolates and the risk factors associated with MRSA colonization among HIV-infected patients has been reported in many countries and regions except mainland China. A systematic review reported that the pooled worldwide prevalence of MRSA in HIV-infected patients was 7%, with the highest prevalence in Southeast Asia (16%) and lowest prevalence in the European region (1%) [5]. It was also found that prior use of antibiotics, recent CD4 count less than 200, and prior hospitalization were general potential risk factors of MRSA colonization for persons who were HIV-positive [6, 7].

Notably, a study suggested that methicillin-resistant coagulase-negative *Staphylococci* (MRCoNS) is a source of the mecA gene, the methicillin-resistance gene, and has the potential contribution to the emergence of MRSA [8]. It also reported that co-colonization of MRSA and MRCoNS isolates in humans may lead to horizontal cross-propagation of resistance genes [9]. In other words, there may be a relationship between MRSA and MRCoNS at molecular level. We therefore hypothesize that there is a possible link between them on population's level, which means MRCoNS carriage may be a potential risk factor of MRSA colonization in HIV-positive patients. However, there is no relevant research on this relationship. Thus, we design this cross-sectional study to explore whether MRCoNS carriage is a risk factor of MRSA colonization in HIV-positive patients.

## 2. Materials and Methods

### 2.1. Study Design and Population

This cross-sectional study was conducted in a large public HIV clinic between June and August 2017. The public HIV clinic, a HIV outpatient clinic of Guangzhou Eighth People's Hospital, has a population of more than 9000 of HIV-infected patients, about 90% of all HIV-infected patients in Guangzhou city. All confirmed HIV-infected patients that aged ≥18 years old and agreed with participation were included in the study, while those who had an acute illness were excluded. The study was approved by the Ethics Committee of Guangdong Pharmaceutical University.

### 2.2. Data Collection

A questionnaire was completed by each enrolled participant after signing an informed consent form. The questionnaire included demographics (age, gender, marital status, and educational level), behavior-related information (smoking status and contact with hay or chaff), details of community-based risk factors (sexual behavior, street drug abuse, and incarceration), medical information (hospitalization, history of influenza, rhinitis, bronchitis, pneumonia, diabetes mellitus, syphilis, and antibiotic use), and HIV-related information (HIV transmission route, CD4 count, and antiretroviral therapy). Nasal samples were collected from both anterior nares of participants using sterile swabs containing sterile saline solution by trained personnel.

### 2.3. Isolation and Identification of MRSA and MRCoNS

Swabs except contacted part were inoculated into 3 mL enrichment broth (Huankai, Guangzhou, China) containing 7.5% NaCl, 1% mannitol, 1% tryptone, and 0.25% yeast extract and incubated at 36 ± 1°C for 24 hours. A loopful of enrichment broth was plated on mannitol salt agar for another 24 hours of incubation. Colonies morphologically suspicious for *Staphylococcus aureus* (*S. aureus*) and CoNS from each mannitol salt agar plate were subcultured to general nutrition agar plates and incubated overnight at 36 ± 1°C. Isolates were identified as *S. aureus* and CoNS based on gram staining, *β*-hemolysis test, catalase test, coagulase test, carriage of 16S rRNA, and nuc genes. Suspicious isolates were identified as *S. aureus* if they were positive for gram staining, *β*-hemolysis test, catalase test, coagulase test, 16S rRNA, and nuc genes. Suspicious isolates were identified as CoNS if they were negative for coagulase test and positive for gram staining, *β*-hemolysis test, catalase test, and the 16S rRNA gene. Detection of 16S rRNA and nuc genes was tested by polymerase chain reaction (PCR) assays [10]. *S. aureus* and CoNS isolates were then, respectively, identified as MRSA and MRCoNS if they were resistant to cefoxitin and positive for the mecA gene [11]. The other *S. aureus* and CoNS isolates were identified as MSSA and methicillin-susceptible coagulase-negative *Staphylococci* (MSCoNS), respectively.

### 2.4. Antimicrobial Susceptibility

Both MRSA and MRCoNS isolates were tested for antibiotic resistance by using Kirby–Bauer disk diffusion method according to the Clinical Laboratory Standards Institute guidelines (CLSI, 2017). Twelve antibiotics were tested, including cefoxitin (FOX: 30ug), penicillin (P: 10unit), erythromycin (E: 15ug), clindamycin (DA: 2ug), tetracycline (TE: 30ug), rifampicin (RD: 5ug), macrodantin (F: 300ug), moxifloxacin (MXF: 5ug), trimethoprim-sulfamethoxazole (SXT: 25ug), teicoplanin (TEC: 30ug), linezolid (LZD: 30ug), and gentamicin (CN: 10ug). Isolates were identified as multidrug resistant (MDR) if they were resistant to ≥ 3 antibiotic classes [12]. The *S. aureus* isolate ATCC25923 was used for quality control.

### 2.5. Molecular Characteristics of MRSA

All MRSA isolates were cultured on general nutrition agar plates at 36 ± 1°C for 24 hours. A single bacterial colony was then inoculated into 3 mL nutritional broth (Huankai, Guangzhou, China) and incubated at 37°C with uniformly vigorous shaking for 16 to 18 hours. Finally, the DNA was extracted using Hipura bacterial DNA kit (Magen, Guangzhou, China) based on the manufacturer's instructions.

Genotypic analysis with pulsed-field gel electrophoresis (PFGE) was performed on all confirmed MRSA isolates, including the Panton-Valentine leukocidin gene (*pvl*) [13], two exfoliating genes (*eta* and *etb*) [14], and the toxic shock syndrome toxin gene (*tst*) [15].

The Staphylococcal cassette chromosome *mec* (SCCmec) typing of MRSA strains were identified as SCC*mec* type I, II, III, IV, and V by using multiplex PCR technique [15]. Those isolates which were not SCC*mec* type I–V were regarded as nontypeable (NT).

The multilocus sequence typing (MLST) PCR assays were performed based on seven housekeeping genes (*arcC, aroE, glpF, gmK, pta, tpiA,* and *yqiL*) as previously described [16]. Sequence types were assigned based on the MLST database (http://www.mlst.net). The clonal complex (CC) was determined by using the eBURST algorithm (http://eburst.mlst.net).

### 2.6. Statistical Analysis

Frequencies and proportions of MRSA colonization in HIV-infected patients were calculated stratifying by characteristics. Analyses of risk factors of MRSA colonization were examined by nonconditional univariable and multivariable logistic regression models. Chi-square test was conducted to examine the differences of phenotypic characteristics between MRSA and MRCoNS isolates. Two-sided *P* values <0.05 were considered statistically significant. All statistical analyses were performed using Stata version 15.1 (Collage Station, Texas, USA).

## 3. Results

### 3.1. Prevalence of MRSA and MRCoNS Nasal Colonization

A total of 1026 HIV-infected patients were eligible to participate and finally 1001 HIV-infected patients were enrolled in this study, including 845 males (84.42%) and 156 females (15.58%). The median age of this population was 35 years (range, 18–81 years). The overall prevalence of *S. aureus* and MRSA colonization among HIV-infected patients was 25.2% (253/1001) and 11.9% (119/1001), respectively. Of the 1001 participants, 575 (57.4%) were colonized CoNS isolates and 423 (42.3%) were colonized MRCoNS isolates. And 41 (4.1%) HIV-infected patients were co-colonized MRSA and MRCoNS isolates.

### 3.2. Risk Factors for MRSA Colonization

In the nonconditional univariate analyses, there were significant differences of MRSA colonization in terms of educational level, smoking status, contact with hay or chaff, history of upper respiratory tract infection within the previous 6 months, history of syphilis within the previous 6 months, the use of HIV antiretroviral therapy, and the status of CoNS colonization. After adjusting the significant factors listed above by using nonconditional multivariate logistic regression model, we found that contact with hay or chaff in the life (adjusted odds ratio [aOR] = 1.79, 95% CI: 1.02–3.15), having a history of syphilis (aOR = 1.77, 95% CI: 1.09–2.90), and suffering from the upper respiratory tract infection within the previous 6 months such as influenza (aOR = 1.64, 95% CI: 1.08–2.49) and rhinitis (aOR = 1.95, 95% CI: 1.14–3.32) were risk factors of MRSA nasal colonization in HIV-positive persons. Current smoking (aOR = 0.58, 95% CI: 0.37–0.90), HIV antiretroviral therapy (aOR = 0.50, 95% CI: 0.27–0.92), and colonization of MRCoNS (aOR = 0.59, 95% CI: 0.38–0.91) were protective factors of MRSA colonization in HIV-positive patients (Table 1).

### 3.3. Antibiotic Resistance

The proportions of antibiotic resistance between MRSA and MRCoNS isolates were significantly different except penicillin, clindamycin, tetracycline, and teicoplanin. Among all MRSA isolates, the most predominant resistant antibiotic was penicillin (89.08%), while the most predominant resistant antibiotic was also penicillin (93.71%) among MRCoNS isolates. Note that the proportion of MDR in MRCoNS isolates was significantly higher than that in MRSA (Table 2).

### 3.4. Molecular Characteristics

**Figure 1:**
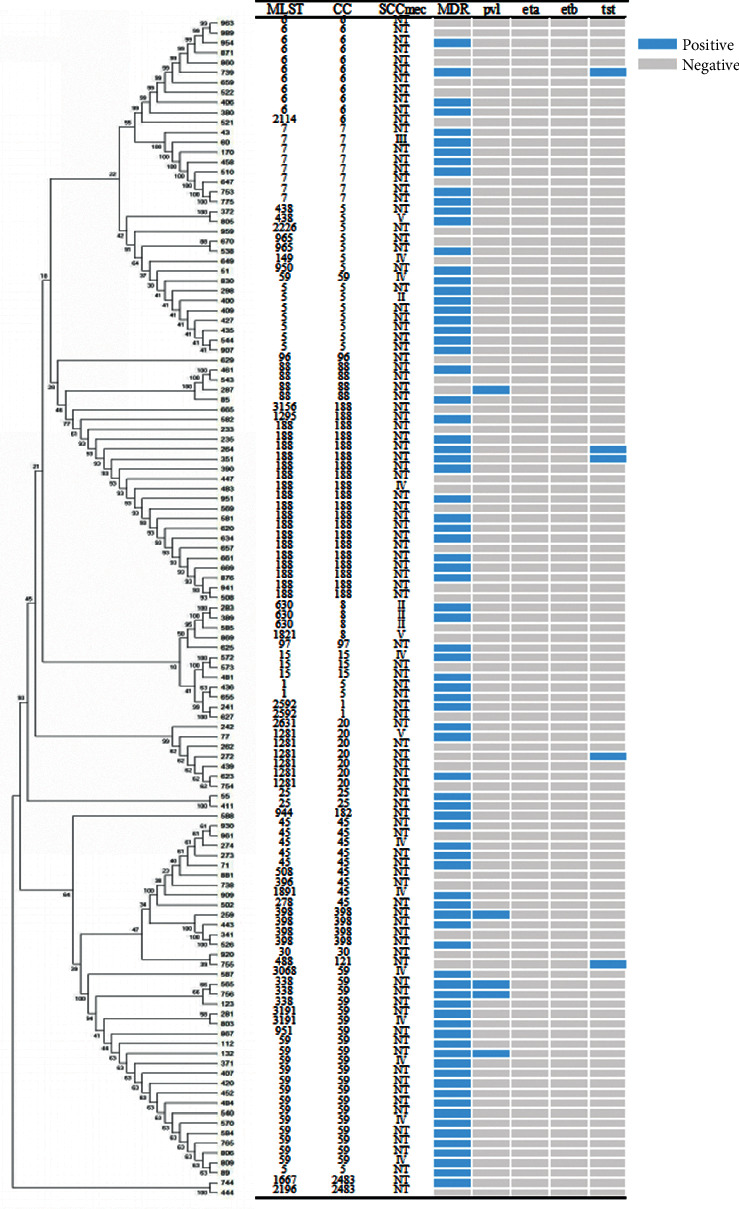
The sequence type and detailed information of MRSA isolates. CC, clonal complex; MLST, multilocus sequence typing; SCC*mec*, staphylococcal cassette chromosome *mec*; NT, nontypeable; MRSA, methicillin-resistant *Staphylococcus aureus*; MDR, multidrug resistance, resistant to no less than three antibiotic classes; *pvl*, Panton-Valentine leukocidin; *eta*, exfoliative toxin A; *etb*, exfoliative toxin B; *tst*, toxic shock syndrome toxin.

Detailed information regarding the molecular characteristics and toxin genes of MRSA is shown in Figure 1. There were 19 CC types and 39 STs in all MRSA isolates in this study. The most predominant ST type among 119 MRSA strains was ST188 (15.1%, 18/119), followed by ST59 (11.8%, 14/119) and ST6 (5.0%, 6/119). The most three predominant CC types among MRSA strains were CC59 (17.6%, 21/119), CC188 (16.8%, 20/119), and CC5 (14.29%, 17/119). In addition, five MRSA isolates were positive for *pvl* gene and five were positive for tst gene. However, no MRSA isolates were positive for *eta* and *etb* genes. Molecular characteristics of MRSA in this study also displayed that 84.03% of MRSA isolates were NT, followed by SCCmec IV (9.2%), SCCmec II (3.4%), SCCmecV (2.5%), and SCCmec III (0.8%).

## 4. Discussion

To the best of our knowledge, this is the first study to explore whether MRCoNS carriage is a risk factor of MRSA colonization. Notably, we found that MRCoNS carriage is a protective factor rather than a risk factor of MRSA colonization. In other words, HIV-infected patients who colonized MRCoNS isolates were less likely to have MRSA colonization.

As for the risk factors of MRSA colonization, we also found that patients who had a history of syphilis were at higher risk for MRSA colonization. This is consistent with a report by Crum-Cianflone et al. [17]. We assumed that this may be caused by lower resistance and immunity in patients with syphilis. It also suggested that MRSA may be transferred during sexual activities and that high-risk behaviors may also increase the risk of MRSA transmission and colonization. Unlike reports of Tilahun et al. [18] and Kyaw et al. [19], we found the history of upper respiratory tract infection rather than lower respiratory tract infection within the previous 6 months was a risk factor of MRSA colonization, which can be associated with the more frequently respiratory episode occurring in HIV-infected population and bacteria colonize in the nasal cavities as the respiratory barrier weakens [20]. Notably, results showed that those who had any contact with hay or chaff were more likely to have MRSA colonization, which was similar to results from Neupane et al. [3]. However, few studies until now investigated the relationships between contact with hay or chaff and MRSA colonization. Similarly, Wang et al. found a significant relationship between smoking status and lower rates of MRSA colonization [21]. In this study, we also found the use of HIV antiretroviral therapy lowered the odds of MRSA colonization as Farley et al. reported [6]. It may suggest that HIV-infected patients using the HIV antiretroviral therapy in primary care clinics could lead to the lower contact with hospitals and then reduce the colonization of MRSA. However, unlike the previous studies [5, 22], there was no significant differences between other factors we investigated and MRSA colonization. The potential reasons for the disparity might be the difference in questionnaire design and characteristics of population in different regions.

The prevalence of MRSA nasal colonization in HIV-infected patients was 11.89%, which was higher than that in a Uganda study (2.41%, 4/166) [7], a Colombian study (1.06%, 2/283) [23], and a study of Taiwan, China (3.44%, 19/553) [24], but lower than that in an American study (15.40%, 77/500) [6] and an Iranian study (12.78%, 23/180) [25]. The prevalence results demonstrated the high burden of MRSA among HIV-infected patients in China, which might be explained by the overuse of antibiotic in the treatment of complications in HIV-infected patients. It also suggested that this population should pay greater attention to nasal hygiene to avoid person-to-person transmission of MRSA isolates. Compared with the previous studies, the differences in prevalence of MRSA might be affected by geological factors, limited sampling locations, and the varying techniques for isolation and identification of bacterial strains.

Among all MRSA isolates, high drug resistance was observed in penicillin, erythromycin, and clindamycin, which is similar to observed studies [4, 26, 27]. In this study, we believe the MRSA and MRCoNS isolates may show differences in all antibiotic resistance. However, opposite to our hypothesis, the antibiotic resistance did not show a statistical difference in penicillin, clindamycin, tetracycline, and teicoplanin. It suggested that there were similarities on the resistance to antibiotics listed above between MRSA and MRCoNS isolates. We supposed the reason was that co-colonization of MRSA and MRCoNS isolates in humans may lead to horizontal cross-propagation of resistance genes as Al-Bakri et al. reported [9]. Further studies are needed to explore the relationship of antimicrobial resistance between MRSA and MRCoNS strains at molecular level. The findings of multidrug resistance of MRSA and MRCoNS isolates in this study were also noteworthy. A significant difference was found between them, in which the multidrug resistant rate in MRCoNS (81.94%) was much higher than that in MRSA (18.06%). This result illustrated that MRCoNS may play an important role in drug resistance. Not only MRSA but also MRCoNS needs to pay more attention to surveillance and monitoring programs.

It was well known that Panton-Valentine leukocidin (*pvl*), a virulent factor encoding toxin, may have a significant influence in some serious *S. aureus* infections, such as severe skin and soft tissue infection and necrotizing pneumonia [28]. We found five (4.20%, 5/119) MRSA isolates were positive for *pvl* toxin gene in this study, which was much lower than a study in Japan (33.33%) [29]. Regarding the virulence genes, another five (4.20%) MRSA isolates were found to be positive for *tst* gene, which was much lower than a study reported among hospital patients (11.60%) [30]. We speculated that the low carrying rate of *pvl* and *tst* genes in the study might be related to the unique characteristics of each population and regional disparity.

A total of 39 STs and 19 CC types of all MRSA isolates were found in our study. Results showed that ST188 was the predominant ST types of MRSA isolates among HIV-infected patients in mainland, China, and ST59 in Taiwan, China [24]. The dominant STs (ST188, ST59 and ST6) in this study were consistent with other community-based populations [31–33], but some other STs were also previously reported in hospital patients (ST338 and ST1) [34–36] and animals (ST398 and ST1) [37–39], which might indicate the cross transmission of isolates in different sources. CC59, the most predominant clonal complex types of MRSA in our study, is the predominant community-associated MRSA clone in Asia [40]. CC5, the third major CC types of MRSA in this study, was reported to be the main CC type of HA-MRSA [41]. The distribution of CC types of MRSA indicated that HA-MRSA strains were gradually infiltrating into the community populations. Based on the distribution of SCC*mec* typing, we found most MRSA isolates in this study were nontypeable, followed by SCCmec IV and SCCmec III. Both HA-MRSA and CA-MRSA were detected in this HIV-positive population, which also suggested that HA-MRSA strains were infiltrating into the community groups.

As the first study to explore whether MRCoNS carriage is a risk factor of MRSA colonization, some limitations should be considered when interpreting the results. Firstly, we can only describe associations between influencing factors and MRSA colonization by using a cross-sectional design, not a causal conclusion. Secondly, we administered the sampling at only one site, which may underestimate the prevalence of MRSA colonization. Thirdly, colonization was only assessed on the day of enrollment. Evaluation of durability of colonization may be useful to fully understand MRSA colonization dynamics.

## 5. Conclusions

HIV-infected patients remain a highly vulnerable population for MRSA colonization, and MRCoNS colonization is a protective factor for MRSA colonization. Moreover, we found similar antibiotic patterns between MRSA and MRCoNS. Further studies of relationship between MRSA and MRCoNS explored at molecular level are needed to examine our hypothesis. Our findings suggested that it is still necessary to pay more attention to MRSA colonization and its antibiotic pattern as well as MRCoNS.

## Figures and Tables

**Table 1:** Analyses of risk factors associated with MRSA colonization in HIV-infected patients [OR (95% CI)].

Characteristics	Non-SA (*N* = 748)	MRSA (*N* = 119)	OR (95%CI)	aOR (95%CI)
Gender (male)	631 (84.36)	107 (89.92)	1.65 (0.88–3.10)	
Age (≥45 years)	202 (27.08)	26 (21.85)	0.75 (0.47–1.19)	
Marital status (married)	351 (47.18)	45 (37.82)	0.68 (0.46–1.01)	
Education (college degree or above)	261 (34.89)	53 (44.54)	1.50 (1.01–2.22)	1.36 (0.90–2.04)
Been arrested	41 (5.48)	9 (7.56)	1.41 (0.67–2.98)	
Current smoker	288 (38.55)	33 (27.73)	0.61 (0.40–0.94)	0.58 (0.37–0.90)
Street drug abuse*∗*	39 (5.28)	9 (7.69)	1.49 (0.70–3.17)	
Contact with hay or chaff*∗*	71 (9.61)	19 (15.97)	1.79 (1.03–3.09)	1.79(1.02–3.15)
Hospitalization*∗*	135 (18.10)	14 (11.76)	0.60 (0.34–1.08)	
History of influenza*∗*	398 (53.71)	76 (64.41)	1.56 (1.04–2.33)	1.64 (1.08–2.49)
History of rhinitis*∗*	79 (10.66)	23 (19.49)	2.03 (1.22–3.38)	1.95 (1.14–3.32)
History of bronchitis*∗*	26 (3.51)	2 (1.69)	0.47 (0.11–2.02)	
History of pneumonia*∗*	27 (3.64)	1 (0.85)	0.23 (0.03–1.68)	
History of antibiotic use*∗*	340 (46.13)	60 (51.28)	1.23 (0.83–1.82)	
History of syphilis*∗*	111 (15.00)	27 (22.69)	1.66 (1.03–2.67)	1.77 (1.09–2.90)
Diabetes mellitus*∗*	19 (2.55)	1 (0.85)	0.33 (0.04–2.49)	
HIV transmission route (sexual)	498 (66.58)	87 (73.11)	1.36 (0.89–2.10)	
HIV antiretroviral therapy	691 (92.38)	103 (86.55)	0.53 (0.29–0.95)	0.50 (0.27–0.92)
CD4 count (<200 cells/uL)	213(28.48)	35 (29.41)	1.05 (0.68–1.60)	
Colonization of CoNS				
No	298 (39.84)	65 (54.62)	Reference	Reference
With MSCoNS	114 (15.24)	13 (10.92)	0.52 (0.28–0.98)	0.52 (0.27–1.01)
With MRCoNS	336 (44.92)	41 (34.45)	0.56 (0.37–0.85)	0.59 (0.38–0.91)

OR, odds ratio; aOR, adjusted OR; CI, confidence interval; SA, *Staphylococcus aureus;* MRSA, methicillin-resistant *Staphylococcus aureus*; MRCoNS, methicillin-resistant coagulase-negative *Staphylococci;* MSCoNS, methicillin-susceptible coagulase-negative *Staphylococci*; CoNS, coagulase-negative *Staphylococci*. *Note.∗*Within the previous 6 months.

**Table 2:** Antibiotic resistance of MRSA and MRCoNS isolates in HIV-infected patients.

Antibiotic (nonsusceptive)	MRSA (*N* = 119)	MRCoNS (*N* = 461)	*χ* ^2^	*P* value
Penicillin	106 (89.08)	432 (93.71)	3.02	0.08
Erythromycin	73 (61.34)	336 (72.89)	6.06	0.01
Clindamycin	50 (42.02)	155 (33.62)	2.92	0.09
Cefoxitin	44 (36.97)	352 (76.36)	67.72	<0.01
Tetracycline	41 (34.45)	161 (34.92)	0.01	0.92
Trimethoprim-sulfamethoxazole	24 (20.17)	250 (54.23)	44.03	<0.01
Moxifloxacin	28 (23.53)	182 (39.48)	10.41	<0.01
Macrodantin	38 (31.93)	30 (6.51)	59.08	<0.01
Teicoplanin	39 (32.77)	113 (24.51)	3.34	0.07
Rifampicin	23 (19.33)	54 (11.71)	4.76	0.03
Gentamicin	7 (5.88)	176 (38.18)	45.68	<0.01
Linezolid	10 (8.40)	9 (1.95)	12.42	<0.01
MDR	82 (18.06)	372 (81.94)	7.73	<0.01

MRSA, methicillin-resistant *Staphylococcus aureus*; MRCoNS, methicillin-resistant coagulase-negative *Staphylococci;* MDR, multidrug resistance, resistant to no less than three antibiotic classes.

## Data Availability

The data used to support the findings of this study are available from the corresponding author upon request.
